# Intelligent Attention-Driven Deep Learning for Hip Disease Diagnosis: Fusing Multimodal Imaging and Clinical Text for Enhanced Precision and Early Detection

**DOI:** 10.3390/medicina62020250

**Published:** 2026-01-24

**Authors:** Jinming Zhang, He Gong, Pengling Ren, Shuyu Liu, Zhengbin Jia, Lizhen Wang, Yubo Fan

**Affiliations:** 1 Medical Engineering & Engineering Medicine Innovation Center, Hangzhou International Innovation Institute, Beihang University, Hangzhou 311115, China; 2 Key Laboratory of Biomechanics and Mechanobiology (Beihang University), Ministry of Education, Beijing Advanced Innovation Center for Biomedical Engineering, School of Biological Science and Medical Engineering, Beihang University, Beijing 100191, China

**Keywords:** hip diseases, deep learning, diagnostic imaging, clinical decision support systems, explainable AI

## Abstract

*Background and Objectives*: Hip joint disorders exhibit diverse and overlapping radiological features, complicating early diagnosis and limiting the diagnostic value of single-modality imaging. Isolated imaging or clinical data may therefore inadequately represent disease-specific pathological characteristics. *Materials and Methods*: This retrospective study included 605 hip joints from Center A (2018–2024), comprising normal hips, osteoarthritis, osteonecrosis of the femoral head (ONFH), and femoroacetabular impingement (FAI). An independent cohort of 24 hips from Center B (2024–2025) was used for external validation. A multimodal deep learning framework was developed to jointly analyze radiographs, CT volumes, and clinical texts. Features were extracted using ResNet50, 3D-ResNet50, and a pretrained BERT model, followed by attention-based fusion for four-class classification. *Results*: The combined Clinical+X-ray+CT model achieved an AUC of 0.949 on the internal test set, outperforming all single-modality models. Improvements were consistently observed in accuracy, sensitivity, specificity, and decision curve analysis. Grad-CAM visualizations confirmed that the model attended to clinically relevant anatomical regions. *Conclusions*: Attention-based multimodal feature fusion substantially improves diagnostic performance for hip joint diseases, providing an interpretable and clinically applicable framework for early detection and precise classification in orthopedic imaging.

## 1. Introduction

With the increasing richness and diversity of medical data, automated disease analysis presents new opportunities while posing formidable challenges for the effective integration of multimodal information. Within musculoskeletal imaging, diagnostic information is typically dispersed across data sources of varying dimensions—such as two-dimensional radiographs, three-dimensional volumetric imaging, and structured and unstructured clinical records. Yet most existing analysis workflows continue to process these data modalities in isolation, failing to fully capture their complementary information and complex cross-modal relationships. This limitation constrains the realization of diagnostic potential.

Hip joint diseases represent a classic scenario illustrating both the value and challenges of multimodal medical analysis. Notably, this study focuses on four clinically prevalent hip joint conditions with substantial diagnostic challenges: hip osteoarthritis (OA), avascular necrosis of the femoral head (ONFH), femoroacetabular impingement syndrome (FAI), and normal hip joints (serving as the control group). These disorders are major contributors to hip pain, functional limitation, and disability across age groups—OA predominantly affects the elderly, ONFH impacts middle-aged adults, and FAI is common in young active individuals [1,2,3]. Their overlapping early symptoms (e.g., groin pain, limited range of motion) and similar radiographic manifestations often lead to misdiagnosis, underscoring the need for a robust diagnostic tool [4]. These diseases present with overlapping early symptoms and complex radiographic manifestations, frequently leading to clinical misdiagnosis [5]. Accurate differentiation is crucial for formulating treatment plans, managing disease progression, and preserving joint function [6]. In routine clinical practice, radiologists typically require integrated assessment of both radiographs and CT scans for diagnostic evaluation [3]. X-rays reveal macroscopic morphological features but have limited capability in displaying subtle lesions; CT scans provide detailed three-dimensional structural information, yet their interpretation often relies heavily on manual analysis and spatial visualization by the clinician. Integrating two-dimensional and three-dimensional information places significant cognitive demand on physicians and is prone to subjective error. Consequently, developing multimodal analysis strategies is regarded as a potential solution to reduce diagnostic uncertainty and enhance clinical efficiency [7].

Moreover, acetabular-femoral impingement syndrome is relatively common, with its imaging features frequently overlapping with those of osteoarthritis and femoral head necrosis [8]. For instance, signals indicative of acetabular labrum damage associated with impingement syndrome are often misinterpreted as cartilage degeneration related to hip osteoarthritis. Such misdiagnoses may lead to inappropriate treatment strategies and significantly increase the risk of patients ultimately requiring total hip arthroplasty [9,10]. Therefore, accurately differentiating degenerative hip disease, avascular necrosis of the femoral head, and impingement syndrome holds significant clinical importance for achieving individualized treatment, controlling disease progression, and preserving hip function.

In the clinical imaging diagnosis of hip diseases, physicians typically need to interpret X-ray, CT images or Magnetic Resonance Imaging (MRI) simultaneously. However, several challenges are posed by this multimodal diagnostic process [11,12]. First, frequent switching between two-dimensional radiographs and three-dimensional CT volumes increases cognitive load, often causing visual fatigue and reducing diagnostic efficiency [13]. Second, although typical degenerative signs such as osteophyte formation and joint space narrowing can be identified on X-ray images, subtle structural and density changes are often present at early disease stages. Reliance on patient history and subjective radiologist interpretation alone easily leads to missed or delayed diagnoses [14]. CT imaging enables three-dimensional quantitative analysis; however, lesions located in non-standard planes are often not captured by conventional axial CT views due to the complex anatomy of the hip joint. Physicians are therefore required to manually adjust reconstruction angles during interpretation [15].

MRI is widely regarded as a reference standard, particularly for early-stage ONFH and soft-tissue evaluation in hip disorders, particularly in early-stage diagnosis. Its superior soft tissue contrast enables visualization of cartilage lesions, acetabular labral tears, marrow edema, and pre-collapse changes in ONFH, which are pathological features often undetectable by X-ray or CT [16]. For FAI, MRI can clearly depict labral pathology and chondral damage associated with bony impingement, while in hip OA, it facilitates early detection of subchondral marrow lesions and synovitis preceding radiographic changes [17]. Additionally, MRI is non-ionizing, making it suitable for serial monitoring in younger patients. However, MRI alone is not universally viable for early hip disease diagnosis due to inherent limitations. First, MRI acquisition is time-consuming (typically 30–60 min), increasing the risk of motion artifacts in elderly or uncooperative patients and reducing diagnostic accuracy [18]. Second, MRI is significantly more expensive, costing 2–5 times as much as X-ray or CT in most settings. This high-cost places it beyond the reach of many patients, especially in resource-limited regions [19]. Third, MRI availability is restricted: only 30–40% of low- and middle-income countries (LMICs) have access to MRI scanners, and even in high-income countries, rural areas often lack this modality [20]. Fourth, patient-related contraindications exclude a significant subset of patients. These include claustrophobia, which affects 5–10% of individuals, metallic implants such as pacemakers and orthopedic hardware, and renal insufficiency when contrast-enhanced MRI is required [21]. Fifth, inter-observer variability in interpreting subtle MRI findings (e.g., early marrow edema, mild labral tears) remains a challenge, particularly among radiologists with limited musculoskeletal expertise [22,23]. These limitations highlight the need for alternative diagnostic approaches that leverage widely accessible modalities to achieve accurate early diagnosis. Overall, existing multimodal interpretation approaches increased the risk of misdiagnosis and made it difficult to efficiently integrate all available imaging information [24].

Machine learning and radiomics approaches have demonstrated feasibility in orthopedic image analysis, exhibiting significant advantages over traditional visual assessment methods. These techniques have been extensively applied to tasks such as disease classification, lesion segmentation, and quantitative assessment, spanning multiple imaging modalities including X-ray, MRI, CT, and ultrasound [25,26,27,28]. However, conventional machine learning and radiomic approaches predominantly rely on domain-knowledge-based manual feature extraction strategies, which to some extent limit their capacity to capture key features from complex imaging patterns [29]. In contrast, deep learning methods can automatically learn features directly from raw image data without requiring manual feature design, thereby demonstrating higher accuracy and robustness when processing large-scale, heterogeneous datasets [30]. Recent studies indicated that certain deep learning models have surpassed experienced clinicians in specific diagnostic tasks. For instance, Peng et al. developed a CT-based deep learning model to predict bone density and perform multi-class diagnosis of normal, osteopenia, and osteoporosis [31]. Validated across multiple hospitals and CT scanners, this model demonstrated robust generalization and clinical applicability. Similarly, Cheng et al. applied an XceptionNet-based model to grade the severity of primary hip osteoarthritis and femoral head necrosis on X-ray images, achieving an AUC of 0.949 and an average sensitivity exceeding that of senior orthopedic specialists [32].

Despite the aforementioned advances, most existing deep learning models remain confined to unimodal imaging inputs, failing to effectively integrate multi-source data such as X-ray films, CT scans, MRI images, and even clinical texts. This limitation constrains the models’ capacity to comprehensively characterize disease patterns and identify complex pathological features [33]. With the advancement of deep learning techniques and the increasing abundance of multimodal data, feature fusion has emerged as a significant research direction within medical artificial intelligence [34,35]. Research indicates that by leveraging complementary information across modalities, multimodal fusion can enhance diagnostic accuracy, robustness, and generalization performance, demonstrating particularly significant effects in scenarios with high pathological heterogeneity [36]. Currently, multimodal deep learning has been extensively explored in oncology and cardiovascular research, yet its application in orthopedic imaging remains relatively underdeveloped [37]. Nevertheless, several exploratory studies have validated the feasibility and potential of multimodal fusion strategies in musculoskeletal applications. For instance, Berk et al. developed a multimodal model integrating hip radiographs, chest radiographs, and clinical data to predict 30-day mortality in elderly hip fracture patients, outperforming traditional scoring systems [38]. Zheng et al. integrated hip and chest CT features with demographic variables, demonstrating that their multimodal fusion model significantly outperformed unimodal approaches, achieving an AUC of 0.914 [39]. Zhou et al. proposed a hybrid deep learning framework based on dual-plane radiographs for bone density prediction and classification, attaining an AUC of 0.970 on an independent test set [40].

To address the above challenges, a unified multimodal deep learning framework was proposed in this study to integrate X-ray images, CT volumes, and clinical texts for automated hip disease classification. The proposed architecture employed modality-specific feature extractors and an attention-based fusion mechanism to model cross-modal interactions and align heterogeneous representations within a unified feature space. By emphasizing system-level design and architecture-oriented fusion rather than disease-specific feature engineering, the framework aimed to improve classification performance and robustness while maintaining extensibility to other multimodal medical imaging tasks.

## 2. Materials and Methods

This section detailed the dataset and methodological workflow employed in this research. The study constructed a multimodal fusion framework designed to collaboratively process three types of medical data: X-ray images, CT scans, and clinical texts. The framework first extracted modality-specific feature representations from each data source via a deep learning-based encoder. Subsequently, a weighted fusion strategy integrated these features to model interactions between cross-modal characteristics. Ultimately, the framework performed a four-class classification task, categorising patients as normal, degenerative hip disease, femoral head necrosis, or acetabular-femoral impingement syndrome.

The overall methodology comprises three principal stages: data preprocessing, construction of the multimodal deep learning framework, and classification performance evaluation. The specific details of each stage will be elaborated upon in subsequent subsections.

### 2.1. Patients

An internal cohort comprising 350 patients (605 hip joints) who visited the Xicheng Campus of Beijing Friendship Hospital between 1 January 2018 and 1 January 2024 was retrospectively collected for model development. In total, 605 hip joints were analyzed, including 163 normal hips, 162 hips with degenerative disease, 140 hips with ONFH, and 140 hips diagnosed with FAI.

An independent and balanced external validation cohort consisting of 15 patients (24 hip joints) from the Tongzhou Campus was included to assess model generalization. This external dataset was entirely independent of the internal cohort originating from the Xicheng Campus. As disease types could differ between the left and right hips, each hip joint was diagnosed and analyzed individually.

Approval for the use of imaging data was obtained from the institutional Ethics Committee (Nos. BFHHZS20230196 and BFHHZS20240058). All X-ray and CT imaging reports were thoroughly reviewed by radiologists. Imaging data were independently assessed by two board-certified radiologists with more than eight years of experience in musculoskeletal imaging. Disease classification was performed according to standardized diagnostic criteria. In cases of disagreement, a third senior musculoskeletal radiologist with over fifteen years of experience adjudicated the findings and determined the final diagnosis.

Patients meeting the following inclusion criteria were enrolled:

(1) availability of an imaging diagnosis corresponding to one of the four categories (normal hip, degenerative hip disease, ONFH, or FAI);

(2) X-ray and CT images covering the anatomical region from the anterior superior iliac spine to the lesser trochanter of the femur.

The exclusion criteria included:

(1) hip fractures, dislocations, or tumors;

(2) prior hip surgery resulting in altered bone structure;

(3) poor-quality or incomplete X-ray or CT images.

All included imaging data were confirmed by radiologists prior to subsequent analysis.

### 2.2. Data Acquisition and Pre-Processing

CT images in the internal cohort were acquired using a PHILIPS (Amsterdam, The Netherlands) BRILLIANCE ICT 256 scanner, whereas CT images in the external cohort were acquired using a Canon (Tokyo, Japan) Aquilion ONE TSX-301CX scanner. X-ray images for the internal cohort were obtained using a CARESTREAM (New York, NY, USA) DRX-Evolution system, while those for the external cohort were acquired using a GE Definium Tempo Pro X-ray system.

All eligible X-ray and CT images were exported from the Picture Archiving and Communication System (PACS) in Digital Imaging and Communications in Medicine (DICOM) format. Clinical text data were also retrieved from the PACS workstation and primarily consisted of pre-imaging clinical information such as patients’ chief complaints, medical histories (e.g., history of steroid use), and physical examination findings. All radiologists’ descriptive content related to X-ray or CT imaging findings was excluded to avoid potential ground truth leakage and ensure the model learns diagnostic features from multi-modal data rather than extracting labels from text.

All hip joints were categorized into four groups: normal, degenerative, necrotic, and FAI by orthopedic and radiology specialists. To avoid data leakage due to inter-hip correlation within the same patient, the dataset was split at the patient level into training, validation, and test sets using a ratio of 7:1.5:1.5. Both hips from the same patient were assigned to the same dataset partition. This ensured independence between training, validation, and test sets, reducing the likelihood that the model learned patient-specific confounders.

Each X-ray and CT image was separated into left-hip and right-hip regions to enable joint-level analysis. All images underwent preprocessing to enhance training efficiency and standardization. Preprocessing steps included format conversion, resolution normalization, and region cropping. Data augmentation techniques were applied to the training set to improve model robustness, including horizontal flipping, random rotation within a range of −10° to 10°, and color jittering [41].

### 2.3. Multimodal Disease Diagnosis Model Development

**Figure 1:**
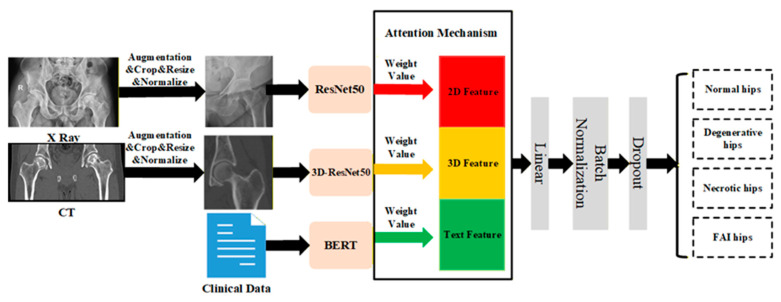
Workflow of the multimodal fusion model for hip joint disease classification.

Table multimodal fusion classification model for hip joint diseases proposed in this study was constructed within a deep learning framework, comprising four core modules: data processing, feature extraction, multimodal fusion, and final classification. The overall workflow of the method was illustrated in Figure 1.

The data processing module was responsible for loading and processing multimodal data. Preprocessing steps included resolution normalization, grey-scale standardization, and intensity calibration to ensure consistency across different imaging modalities and acquisition devices. For images data, the Region of Interest (ROI) was defined as the anatomical range from the anterior superior iliac spine to the lesser trochanter to ensure the anatomical consistency of the analyzed region and reduce interference from irrelevant background. The ROI was manually cropped following a standardized operation protocol (SOP). Two board-certified musculoskeletal radiologists with 8 and 12 years of clinical experience, respectively, performed the manual cropping. In cases where the two readers disagreed, a third senior musculoskeletal radiologist (over 15 years of experience) adjudicated the findings and made the final decision on ROI boundaries.

To assess inter-observer consistency of the manual ROI cropping process, 20 randomly selected cases were independently processed by the two radiologists. For CT volumes, inter-observer agreement of the cropped ROIs was quantified using the Dice Similarity Coefficient (DSC), yielding a mean DSC of 0.94 (95% CI: 0.91–0.96), indicating spatial consistency and reproducibility. X-ray ROIs were cropped according to the same anatomical guidelines, and visual consistency between readers was confirmed. After ROI definition, CT data were converted from DICOM to NIfTI format, and X-ray images were exported as PNG files. X-ray images were resized to 224 × 224 pixels, normalized using Z-score standardization, and replicated across three channels to match the input requirements of the ResNet50 backbone. All manually cropped CT volumes were resampled to a uniform voxel spacing of 1.0 × 1.0 × 1.0 mm^3^ using trilinear interpolation to eliminate scanner-specific variations. To balance computational efficiency and preservation of clinically relevant anatomical features, the resampled CT volumes were downsampled by a factor of 2 along the z-axis (resulting in a final voxel spacing of 1.0 × 1.0 × 2.0 mm^3^). A pilot study confirmed that this strategy was empirically observed to preserve key bony structures relevant for diagnosis [42]. A Gaussian filter with σ = 0.5 was applied to reduce soft tissue artifacts and background noise while preserving diagnostic bony details. After preprocessing, each CT volume was resized to a fixed input size of 128 × 128 × 64 voxels before being fed into the 3D-ResNet50.

The feature extraction module independently extracted representative features from CT images, X-ray images, and clinical text. The preprocessed 3D CT data were then fed into the 3D-ResNet50 network—comprising an initial convolutional layer, four residual blocks, and a global average pooling layer—to effectively extract three-dimensional structural features [43]. For two-dimensional X-ray images, we employed a ResNet50 architecture pre-trained on ImageNet. This comprised an initial convolutional layer, multiple residual stages (conv2x to conv5x), and fully connected layers, enabling hierarchical extraction of image discriminative features [44]. Clinical text features were extracted using a pre-trained BERT model. The BERT architecture stacks multiple Transformer encoder layers, each incorporating self-attention mechanisms and feedforward neural networks, enabling effective modelling of context-dependent relationships within radiology reports and clinical descriptions [45].

The multimodal fusion module employed an attention-based fusion strategy to integrate heterogeneous feature representations from different modalities. This module dynamically assessed the relative importance of each modality by assigning adaptive weights between 0 and 1. Modalities with higher feature quality and stronger relevance were assigned greater weights, while those with less informative content contributed correspondingly smaller weights. Ultimately, by weighting and integrating modality-specific features, a fused feature representation was obtained, enabling effective cross-modal information aggregation [46].

The classification module received the fused feature representation and output a four-class prediction corresponding to normal hip joints, degenerative hip disease, femoral head necrosis, and femoroacetabular impingement syndrome. To ensure experimental reproducibility, the random seed was fixed during training. The model training batch size was set to 8, with parameter optimization employing the Adam optimizer. A cosine annealing learning rate scheduler was applied to enhance training stability, with a maximum learning rate of 0.001 [47]. The optimization objective utilized the cross-entropy loss function, incorporating L2 regularization and stochastic dropout techniques to mitigate overfitting. The model underwent 200 training epochs.

All experiments were implemented in Python 3.10 using the PyTorch 1.10.0 framework. Training was conducted on a workstation running Windows 11 Pro, configured with 128 GB RAM, an Intel i9-14900K CPU (3.2 GHz), and an NVIDIA GeForce RTX 4070 Super GPU with 16 GB VRAM.

### 2.4. Model Analysis and Validation

To evaluate the classification performance of the trained multimodal fusion model, we first assessed its ability to correctly categorize samples into four disease classes (normal, degenerative lesions, necrotic lesions, and impingement syndrome) on an internal test set. Concurrently, to further evaluate the model’s generalization capability across different clinical centers and imaging modalities, supplementary validation was conducted on an independent external validation cohort.

Model performance was primarily quantified using receiver operating characteristic curves and their area under the curve. Furthermore, we generated confusion matrices and calculated quantitative metrics including accuracy, sensitivity, and specificity to comprehensively evaluate the model’s classification efficacy.

To enhance model interpretability and analyze its decision-making basis, we employed gradient-weighted class activation mapping (Grad-CAM). This visualization technique highlighted key image regions contributing most significantly to model predictions, thereby facilitating a clinically grounded qualitative assessment of the relevance and reliability of model decisions [48].

## 3. Results

### 3.1. Demographical Characteristics

This study included 350 patients, encompassing 605 hip joints. The cohort’s mean age was (66.3 ± 12.3) years. Detailed demographic characteristics of the overall dataset are summarized in Table 1. The dataset was partitioned into training, validation, and test sets. The training set comprised 423 cases (114 normal, 113 degenerative, 98 necrotic, 98 impingement syndrome), the validation set contained 90 cases (24 normal, 24 degenerative, 21 necrotic, 21 impingement syndrome), and the test set comprised 92 cases (25 normal, 25 degenerative lesions, 21 necrotic lesions, 21 impingement syndromes). Baseline characteristics for each subset are presented in Table 2. Statistical analysis revealed no statistically significant differences between the training, validation, and test sets (*p* > 0.05), indicating appropriate data partitioning.

### 3.2. Training Dynamics Analysis

**Figure 2:**
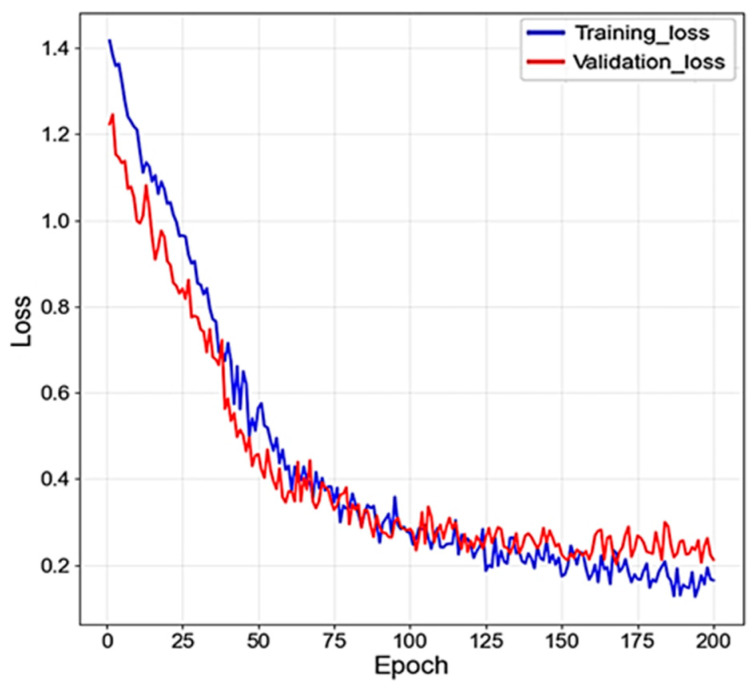
Loss curves of the training process.

The multimodal fusion model was trained for 200 epochs with a learning rate of 0.0001 and a batch size of 8. The training and validation loss curves are shown in Figure 2. Both curves decreased steadily during training and followed similar trends, suggesting stable convergence. No evident divergence between training and validation losses was observed, indicating that the model maintained acceptable generalization performance without obvious overfitting.

### 3.3. Classification Performance on the Internal Test Set

**Figure 3:**
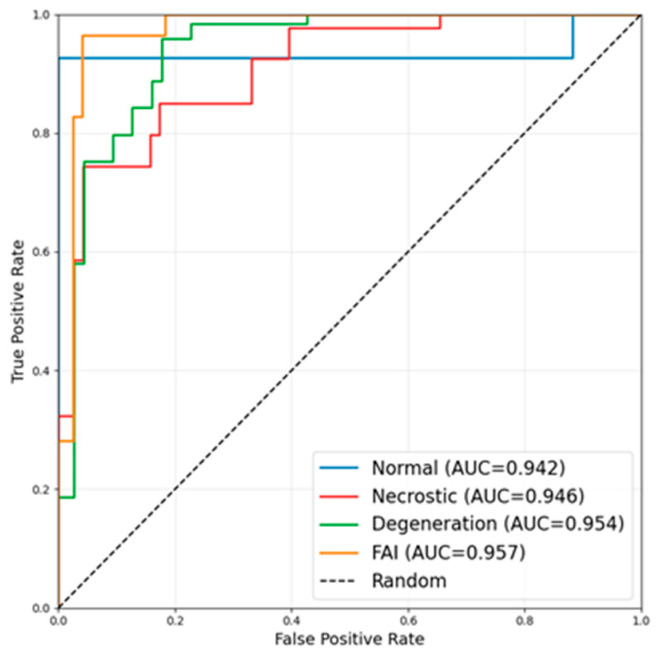
The ROC curves of the classification model.

Classification performance on the internal test set is summarized in Table 3 and Figure 3. Precision and recall were key metrics for assessing classification performance [49]. High precision and recall were achieved across all four categories. The normal group achieved a precision and recall of 0.920, indicating accurate identification of normal hips with minimal confusion. The necrotic group demonstrated the highest recall (0.952), suggesting that most ONFH cases were successfully detected, although precision was slightly lower (0.909), reflecting limited misclassification with non-necrotic cases. The degenerative group achieved balanced precision (0.920) and recall (0.920), while the FAI group showed high precision (0.950) with relatively lower recall (0.905), indicating a small proportion of missed FAI cases.

Sensitivity and specificity were key metrics for evaluating model performance [50]. As shown in Table 3, sensitivity and specificity exceeded 0.900 across all disease categories. Classification performance was particularly outstanding between the normal and necrotic groups, achieving sensitivities of 0.920 and 0.952, respectively, with specificities of 0.970 and 0.986. Sensitivities were marginally lower for the degenerative disease and impact syndrome groups, indicating a small number of missed diagnoses; however, both groups maintained high specificities. Receiver operating characteristic curve analysis revealed AUC values were consistently high across all categories, reflecting the model’s excellent discriminatory capability.

**Figure 4:**
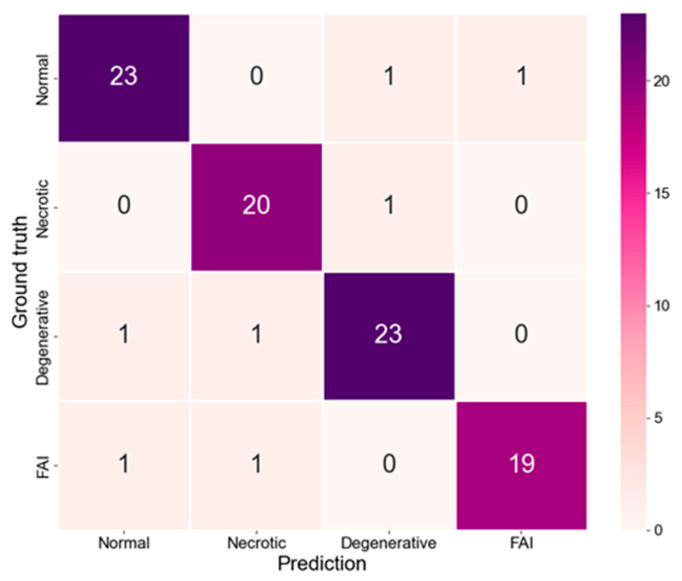
Confusion matrices of the classification model.

The confusion matrix depicted in Figure 4 further elucidates the model’s classification performance. Results indicated that the majority of cases were correctly classified across all categories. Misclassifications were predominantly concentrated among degenerative lesions, necrotic lesions, and impingement syndrome, which is consistent with the partial overlap in their imaging characteristics. Overall, the model demonstrated optimal performance in identifying normal hips while maintaining reliable discrimination capabilities across different pathological subtypes.

### 3.4. Comparative Analysis of Different Modality Fusion Strategies

To clarify the marginal gain of multimodal fusion compared to single-modality image analysis, seven additional baseline models were constructed: (1) “X” model (using only X-ray images as input); (2) “CT” model (using only CT images as input); (3) “Clinical” model (using only clinical text as input); (4) “Clinical+X” model (using clinical text and X-ray images as input); (5) “X+CT” model (using X-ray images and CT images as input); (6) “Clinical+CT” model (using clinical text and CT images as input); (7) “Clinical+X+CT” model (using clinical text, X-ray images and CT images as input). The performance metrics of these baseline models on the internal test set are summarized in Table 4.

**Figure 5:**
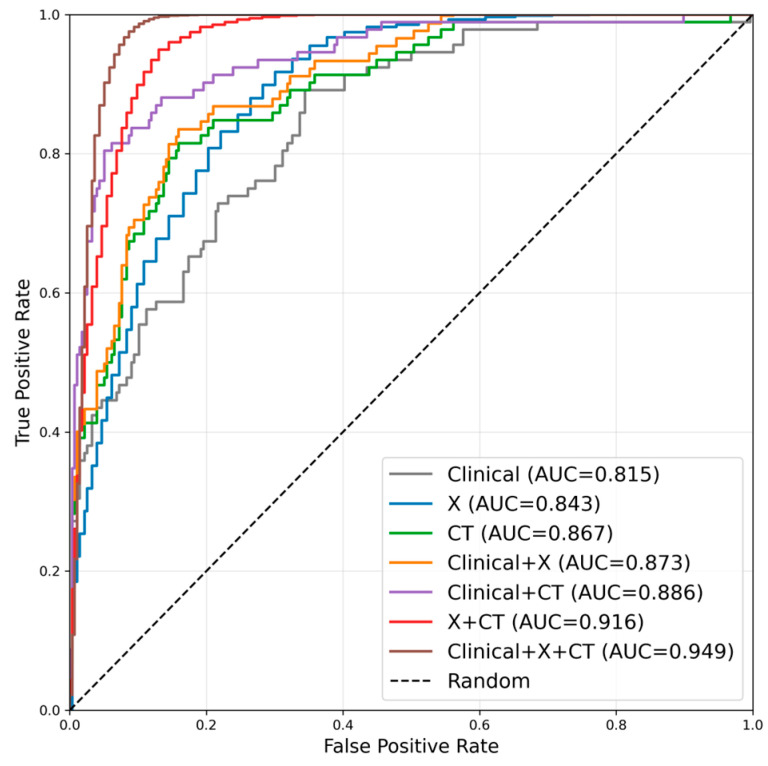
ROC curves comparison of different modality fusion strategies.

Receiver operating characteristic (ROC) analysis was employed to compare the classification performance of different multimodal fusion strategies and image-only baselines for hip joint disease diagnosis. The ROC curves represented macro-averaged one-vs-rest performance across four classes. As summarized in Table 4 and Figure 5, the X model achieved an AUC of 0.843, and the CT model achieved an AUC of 0.867. Both image-only baselines outperformed the Clinical-Only model (AUC = 0.815), but were surpassed by the multimodal fusion strategies: Clinical+X (AUC = 0.873), Clinical+CT (AUC = 0.886), X+CT (AUC = 0.916), and the fully integrated Clinical+X+CT model (AUC = 0.949).

Notably, the Clinical+X+CT model outperformed the best-performing image-only baseline (CT-Only, AUC = 0.867) by an 8.0% absolute increase in AUC, demonstrating a clear and substantial marginal gain from multimodal integration. The X+CT model (AUC = 0.916) also consistently exceeded individual image-only baselines, confirming the complementary value of combining X-ray and CT data. Furthermore, the Clinical+CT model (AUC = 0.886) outperformed the CT model (AUC = 0.867), and the Clinical+X model (AUC = 0.873) surpassed the X model (AUC = 0.843), indicating that clinical text added unique diagnostic information that enhances the performance of single imaging modalities.

In terms of other key metrics, the Clinical+X+CT model achieved the highest sensitivity (0.924), specificity (0.978), precision (0.924), and F1-score (0.924) among all strategies. The X+CT model followed with solid performance (AUC = 0.916, F1-score = 0.902), while the single-modality models showed relatively lower but still acceptable performance.

**Figure 6:**
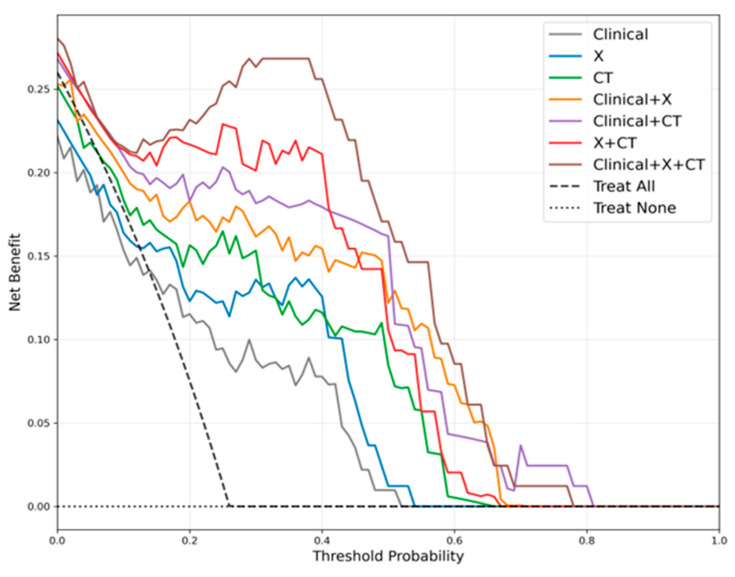
DCA curves comparison of different modality fusion strategies.

Decision curve analysis (Figure 6) further confirmed the clinical utility of multimodal fusion: the Clinical+X+CT model maintained the highest net benefit across a wide range of clinically relevant thresholds in this dataset, followed by the X+CT model, CT model, Clinical+CT model, Clinical+X model, X model, and Clinical model. This indicated that integrating clinical text with imaging data reduces unnecessary interventions and diagnostic errors more effectively than standard image-only analysis or clinical text alone.

**Figure 7:**
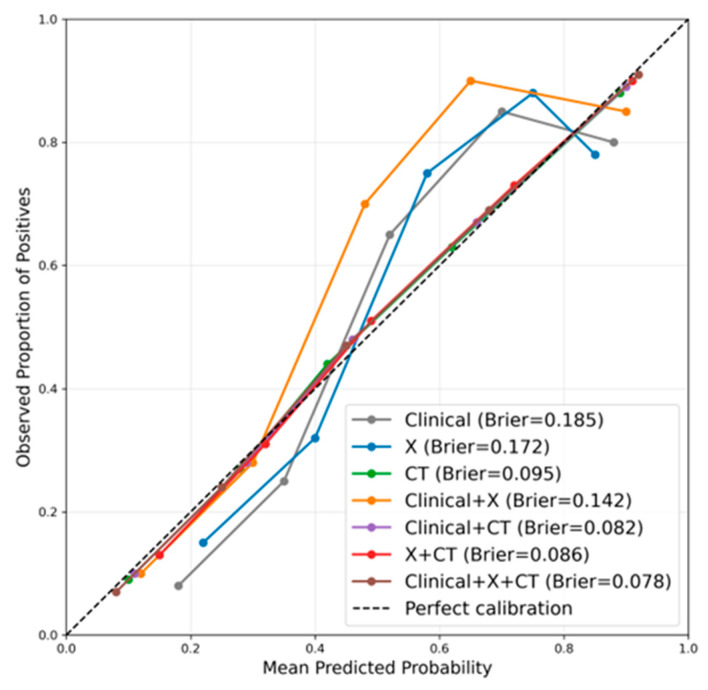
Calibration curves comparison of different modality fusion strategies.

Calibration curves were used to assess the agreement between predicted probabilities and observed outcomes (Figure 7). All models exhibited acceptable calibration performance, with the Clinical+X+CT model showing the closest alignment between predicted and observed probabilities (Brier score = 0.078). The X and CT models demonstrated slightly worse calibration (Brier scores: 0.172, 0.095) than the multimodal models, suggesting that clinical text helped refine probability estimates and improve predictive reliability.

### 3.5. Grad-CAM-Based Model Interpretability Analysis

**Figure 8:**
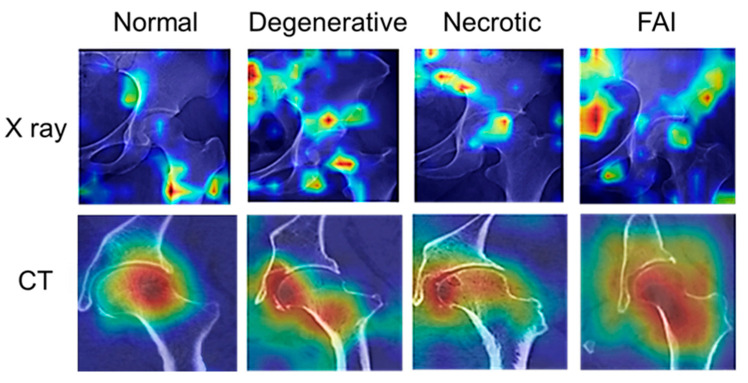
Grad-CAM visualization results for four hip joint disease groups.

Gradient-weighted class activation mapping (Grad-CAM) was applied to visualize image regions contributing most to the model’s predictions. Representative heatmaps for four high-confidence cases were shown in Figure 8. In these visualizations, regions with higher attention were highlighted in red.

For the normal group, attention primarily focused on the pelvis and proximal femoral regions in X-ray images, and on the femoral head and acetabular surface in CT images. In the degenerative group, attention was concentrated around the femoral head–acetabulum junction and the lower femoral neck on X-ray images, with corresponding focus on the medial femoral head and femoral neck edges on CT images. For the necrotic group, the model consistently attended to the femoral head regions associated with necrotic lesions in both X-ray and CT images, particularly in weight-bearing areas. In the FAI group, attention patterns were more dispersed, covering the femoral neck, acetabulum, and proximal femoral structures.

Overall, the attention maps demonstrated that the model relied on anatomically and clinically relevant regions during decision-making, supporting the interpretability and rationality of the proposed multimodal framework.

### 3.6. External Validation Results

To address the potential limitation of small sample size in the external validation set, we performed bootstrapping analysis with 1000 iterations to assess the stability of the model’s performance. This method generates multiple resampled datasets from the original external validation cohort, enabling the calculation of 95% confidence intervals (CIs) for all key metrics. The results offered a transparent quantification of performance variability under small-sample conditions.

The multimodal fusion model with the best internal performance was further evaluated on an independent external validation set (n = 24). As summarized in Table 5, five of six normal cases and five of six necrotic cases were correctly classified, with the remaining cases in both groups misclassified as degenerative. In the degenerative group, four of six cases were correctly identified, while one case was misclassified as normal and another as FAI. All FAI cases were correctly classified.

The external validation results demonstrated consistently high diagnostic performance across most categories. The AUC values remained high for all four classes (Normal: 0.950; Necrotic: 0.965; Degenerative: 0.880; FAI: 0.980), with corresponding 95% CIs indicating limited variability despite the small cohort size. For sensitivity and specificity, the estimated confidence intervals reflected moderate uncertainty, particularly in the degenerative group, which is consistent with the observed inter-class overlap and small sample size.

Overall, no misclassifications were observed in the current external cohort in the FAI group and the model maintained high sensitivity and specificity in the normal and necrotic groups, while comparatively lower performance was observed for degenerative cases. These findings indicate that the proposed model preserved stable and clinically acceptable classification performance on data acquired from a different center and imaging systems. These results provided preliminary evidence of cross-center feasibility.

## 4. Discussion

Early detection of hip joint degenerative disease and ONFH is a challenge for clinicians and surgeons. Previous studies reported low interobserver agreement among clinicians when diagnosing early-stage ONFH, indicating that such assessments were largely subjective [51]. In this study, a multimodal feature fusion classification model based on deep learning, integrating clinical texts, X-ray images and CT images, was proposed and validated to assist clinicians in accurate classification of hip joint diseases. By capturing complementary information from different modalities, both recognition accuracy and generalization ability were consistently enhanced by the method. Among the different fusion strategies, the “Clinical+X+CT” model achieved the best performance, with AUC values close to 1.0 (0.942–0.957) across the “normal”, “degenerative”, “necrotic”, and “FAI” groups. Moreover, its precision (0.909–0.950), recall (0.905–0.952), and F1-scores (0.920–0.930) consistently remained at high levels on the test set.

The recognition performance of different categories in the multimodal feature fusion classification model was influenced by imaging characteristics, specificity of clinical information, and case size. This led to variations across performance metrics. For the “normal” group, both sensitivity (0.920) and specificity (0.970) were high, and precision was the highest among all groups. This indicated that normal cases were relatively well separated within the current dataset from FAI categories in both imaging and text features. On X-ray and CT images, normal hip joints typically showed uniform joint space, continuous bone structure, and no local abnormalities. These features created distinct and independent features in the multimodal feature space, making them easier for the model to identify [52]. The “necrotic” group achieved the highest recall (0.952) with an AUC of 0.946, indicating strong sensitivity for detecting ONFH. This was due to clear density changes in necrotic lesions on CT images, such as cystic or collapsed regions, combined with distinctive clinical records (e.g., long-term corticosteroid use, persistent hip pain). The integration of these multimodal features formed stable discriminative patterns [53]. However, precision was slightly low (0.909), showing that some degenerative or FAI lesions were misclassified as ONFH category. This may result from local bone changes or irregular morphology, a challenge also noted in previous multiclass studies. The precision in this study was still higher than most single-modality recognition models [54,55,56]. For the “degenerative” group, precision was relatively high (0.920). This suggested that the model could accurately identify degenerative cases, though some were misclassified. Early-stage degeneration showed subtle imaging features, and clinical symptoms may be atypical. Additionally, overlaps between degenerative changes and ONFH (e.g., acetabular edge sclerosis, joint space narrowing) may complicate differentiation [57]. The “FAI” group had a precision of 0.950, but a low recall of 0.905, reflecting insufficient detection of these cases. This may be due to the relatively small sample size, which limited the model’s ability to learn representative features [58].

Performance differences across fusion strategies exhibited a clear progressive pattern, fully reflecting the complementary value of multimodal information. The single clinical text model (Clinical) integrated only symptoms, physical signs, and pre-imaging clinical variables, which were purified to exclude imaging descriptions. These features were mostly indirect clinical manifestations, lacking direct representation of joint morphology or bone density changes. As a result, its diagnostic performance was relatively limited, with an AUC of 0.815. This finding aligns with previous studies: models relying solely on clinical text or structured electronic medical record data often have insufficient discriminative power for orthopaedic diseases—especially when imaging findings are subtle or symptoms are nonspecific—and such models are prone to underdiagnosis or misclassification [59]. Adding X-ray images (Clinical+X) introduced macroscopic morphological information, including gross bone structure, joint space width changes, and osteophyte formation—features critical for identifying hip degeneration, deformity, and necrosis. As a low-cost, widely accessible modality (compared to CT and MRI), X-ray has long been confirmed as a core tool for hip disease screening [60,61]. In our study, integrating X-ray features improved the AUC from 0.815 (Clinical) to 0.873 (Clinical+X), highlighting the complementary effect of macroscopic imaging features and clinical text context. Further integrating CT images (Clinical+CT) elevated performance to an AUC of 0.886. CT provided higher spatial resolution than X-ray, clearly revealing cortical thickness, subcortical structural changes, and lesion boundaries—detailed that enhanced the identification of subtle pathological features (e.g., early subchondral sclerosis in necrosis) [62]. This aligned with prior reports that CT features improved the sensitivity and specificity of AI-assisted musculoskeletal diagnosis, especially for multi-class tasks [63]. The combination of X-ray and CT (X+CT) achieved an AUC of 0.916, which was consistently higher than either single imaging modality (X: 0.843; CT: 0.867). This confirmed that X-ray (macroscopic morphology) and CT (fine structural details) are highly complementary: X-ray provides a rapid overview of joint status, while CT supplements high-precision anatomical information, together forming a more comprehensive imaging feature set.

Finally, the fully integrated Clinical+X+CT model achieved the best performance, with an AUC of 0.949 (the highest among all strategies) and a specificity of 0.978. This result verified that the tripartite fusion of “clinical context + macroscopic morphology (X-ray) + fine structure (CT)” maximized information complementarity: clinical text provides patient background (e.g., steroid use history for necrosis), X-ray offered initial morphological screening, and CT supplies detailed pathological evidence. This multi-layered information integration not only improved the recognition accuracy of individual categories but also enhanced inter-class discrimination—few misclassifications between “normal”, “necrotic”, and “degenerative” groups were observed in the confusion matrix, particularly in distinguishing degeneration from necrosis. CT’s 3D bone structure and lesion margin information enabled the model to capture subtle disease differences and reduce category overlap, a pattern consistent with multimodal studies in oncology and cardiovascular diseases [64,65,66]. The cause of performance differences lay in the complementarity of each modality [67]. Patient history and symptom context were provided by clinical text, while macroscopic structural information was offered by X-ray images, and detailed three-dimensional bone features were added by CT images. Together, high accuracy and stability were achieved by the multimodal fusion classification model through these modalities.

The external validation outcomes reflect the model’s generalizability and reveal characteristic patterns of cross-cohort diagnostic performance. For normal hips and FAI, the model demonstrated consistently high classification accuracy, which aligns with prior multi-center musculoskeletal AI studies. These categories are characterized by relatively distinct and stable structural features, facilitating reliable feature transfer across imaging systems and reducing cross-cohort variability [68,69,70]. Osteonecrosis similarly maintained high diagnostic reliability in the external cohort, consistent with its well-defined subchondral CT signatures that are less sensitive to population-specific variation. This observation aligns with previous ONFH diagnostic models, in which CT-derived features have been shown to contribute to stable cross-center performance. In contrast, comparatively lower performance was observed in the degenerative group. This finding is consistent with the inherent heterogeneity of hip degeneration, where the extent of joint space narrowing, osteophyte formation, and subchondral alterations varies substantially across populations and imaging conditions. Previous studies have likewise reported greater cross-cohort performance variability in degenerative disease models compared with conditions characterized by more focal and distinct lesions, such as FAI or ONFH [71]. Despite the limited sample size of the external validation cohort, the estimated confidence intervals for key performance metrics remained within a clinically acceptable range, and no systematic degradation of performance was observed across categories. These results suggest that the integration of multimodal features, combining clinical information with complementary imaging modalities, may help mitigate modality-specific noise and enhance model robustness across centers.

Comparatively, the model’s external performance for focal lesions (FAI, ONFH) matches or exceeds existing hip AI models (which often report external AUCs < 0.9 for these categories), while its degenerative group performance is consistent with benchmarks for heterogeneous orthopedic conditions. The variation across categories underscores that diagnostic stability correlates with the consistency of disease-specific imaging features, a pattern observed in other musculoskeletal multimodal models. It was shown by Grad-CAM heatmaps that anatomical and pathological regions closely related to clinical diagnosis (Figure 6). In “normal” group, the pelvis, the upper femoral neck, and the joint space were highlighted by the X-ray heatmaps. Cortical continuity and smooth joint surfaces were mainly covered by the CT heatmaps. These regions were key indicators for evaluating joint contour integrity and joint space width, both of which reflect overall joint health [72,73]. In the “degenerative” group, the sacrum, ischium, and the connection between the femoral head and acetabulum were the main focuses of the X-ray heatmaps, while irregular joint surfaces and osteophyte formation were the main focuses of the CT heatmaps. These findings were crucial radiographic features for assessing the severity of osteoarthritis [74,75]. In the “necrotic” group, the weight-bearing areas of the femoral head and the subchondral lesion regions were the main focuses of the model. These areas bore major loads and were vulnerable to vascular impairment. They were also the initial sites of morphological and density changes in early ONFH and represented critical regions for CT-based diagnosis [76,77,78]. The “FAI” group included cases of femoroacetabular impingement. The sacrum, ilium, iliac wing, and parts of the lateral femoral head were highlighted by the X-ray heatmaps. The femoral neck, proximal femoral shaft, greater and lesser trochanters, and the acetabulum were emphasized by the CT heatmaps. These findings suggested that the model focused on insufficient acetabular coverage and femoral head displacement, which were key signs of femoroacetabular impingement. They also corresponded to sites prone to osteophyte formation and labral damage [79,80]. Overall, the image regions highlighted by the model were consistent with clinical diagnostic logic, enhancing its credibility. The distribution of hot spots differed among disease categories. In normal cases, hot spots were relatively concentrated and symmetric, mainly reflecting bone integrity. In degenerative disease, strong hot spots appeared around load-bearing areas and the joint space, indicating that the model captured features of cartilage wear and osteophyte formation. In the “necrotic” group, hot spots clustered in the superior weight-bearing region of the femoral head and the edges of collapse, reflecting bone structural failure and local density abnormalities. In FAI hip diseases, hot spots appeared dispersed, often at sites of abnormal morphology or density. These findings suggested that the model relied on these differences for classification. It was indicated by this characteristic distribution of hot regions across categories that the model’s decision-making mechanism adapts its focuses according to the disease types. Grad-CAM was an intuitive visualization tool that made the decision process transparent to clinicians [48]. In multimodal diagnostic tasks, interpretability was particularly important. The model’s basis was quickly understood by clinicians, and trust in AI-assisted results was improved [32]. In this study, different focus patterns between X-ray and CT modalities were clearly demonstrated by the heatmaps. Overall bone contour and joint space changes were captured by X-ray images, while cortical discontinuity and lesion areas were more sensitively detected by CT images. These differences not only verified the value of multimodal fusion, but also suggested a feasible path for integrating the model into clinical workflows.

The clinical implications of the proposed “Clinical+X+CT” model extend beyond conventional imaging interpretation. First, it reduces the cognitive burden on radiologists by automatically integrating and analyzing multimodal data, minimizing the need for manual switching between X-ray and CT images and reducing visual fatigue [81]. Second, the Grad-CAM visualization provides interpretable evidence for diagnostic decisions, allowing radiologists to quickly verify the model’s focus on clinically relevant anatomical regions (e.g., weight-bearing areas of the femoral head in ONFH) and enhancing trust in AI-assisted results. Third, the model improves the efficiency of early diagnosis, especially for subtle lesions that may be missed by subjective human interpretation, which is critical for hip-preserving treatments and reducing the risk of total hip replacement [82,83].

In low- and middle-income countries (LMICs), where access to advanced imaging modalities such as MRI is limited due to high costs and inadequate infrastructure [20], the proposed model offers a practical alternative. X-ray and CT are more widely available and cost-effective in these settings, and the model’s high diagnostic performance (AUC = 0.949) enables accurate classification of hip diseases without relying on MRI. This can consistently improve access to quality diagnostic services for underserved populations, reducing health disparities in orthopedic care.

Regarding the role of MRI in hip joint evaluation, the proposed model complements MRI rather than replacing it in scenarios where MRI is accessible. MRI remains irreplaceable for assessing soft-tissue abnormalities (e.g., labral tears in FAI) and early marrow edema in ONFH [80]. However, the model provided a reliable substitute when MRI is unavailable, contraindicated, or unaffordable. For example, in patients with claustrophobia or metallic implants, the model can accurately classify hip diseases using X-ray, CT, and clinical text, avoiding the need for MRI. Additionally, the model’s ability to integrate clinical text with imaging data helped compensate for the lack of soft-tissue information in X-ray and CT, further enhancing its diagnostic value in the absence of MRI.

Multimodal fusion has shown significant advantages in orthopaedic imaging diagnosis. By combining data from different sources, models can integrate macroscopic bone structure, lesion details, and clinical features, thereby improving predictive performance. For example, Berk et al. developed a model that fused hip X-rays images, chest X-rays images, and clinical information (including height, weight, mobility assessment, and comorbidities) to predict the 30-day mortality risk in elderly patients with hip fractures. The results showed that this model outperformed traditional scoring methods, with chest X-ray images and clinical data contributing most to its performance [38]. Similarly, Zheng et al. fused hip and chest CT imaging features with clinical variables such as sex and age, significantly improving the accuracy and reliability of osteoporosis prediction. Using clinical data alone yielded an AUC of only 0.601, whereas the fusion model achieved an accuracy of 0.915 and an AUC of 0.914 [39]. In addition, Zhou et al. proposed a deep learning framework that combined features from dual-plane X-rays images for bone mineral density prediction and classification. Its diagnostic performance was highly consistent with QCT-based bone density measurements, reaching an AUC of 0.970 on an independent test set, highlighting the potential of low-radiation, low-cost screening [40]. It was demonstrated by these studies that multimodal fusion can effectively integrate imaging and clinical information. Diagnosis and prediction that were more precise and reliable than those achieved by single-modality approaches were thereby enabled. Compared with previous deep learning and multimodal studies in orthopaedics, an AUC of 0.949 was achieved by the “Clinical+X+CT” multimodal feature fusion classification model. And the values close to 1 were maintained across degenerative and FAI diseases, indicating strong stability and superiority in multiclass recognition. In decision curve analysis (DCA), the highest net benefit across most threshold ranges was also provided by the model. From the perspective of information complementarity, history and symptom features were contributed by clinical text, providing important clues when imaging findings were atypical. Rapid localization of potential lesion areas was allowed by X-rays images, providing a first-line tool. Decisive support was provided by CT images through the revelation of structural details of lesions. Previous studies often focused on only one or two sources of information, whereas maximal integration of all three sources was achieved by our multimodal fusion strategy. As a result, both high accuracy and strong robustness in multiclass tasks were achieved by the multimodal feature fusion classification model.

## 5. Limitations and Prospects

This study had several limitations. First, due to the specific data requirements, paired X-ray and CT images were needed for each patient, the sample size remained relatively small. Larger-scale external validation was therefore necessary. In this study, the paired design helped improve data consistency and reduce variability. This approach also highlighted the value of multimodal fusion in hip disease diagnosis and suggested a feasible path for integrating AI models into clinical workflows [84,85]. Second, the diagnostic standards for hip joint diseases in this study were based on X-ray, CT imaging, and clinical text descriptions, without histopathological confirmation. Nevertheless, the combination of imaging and medical records was a widely accepted strategy in both clinical practice and previous research, and thus retained clinical value [86]. Third, the current model integrated only X-ray and CT features. In practice, MRI was also important for diagnosis, but data availability limited its inclusion in this study. Future multicenter collaborations will be needed to ensure adequate MRI data. Despite this limitation, focusing on X-ray and CT features aligned well with routine examinations in most hospitals and enhanced the feasibility of clinical application [87].

## 6. Conclusions

A “Clinical+X+CT” multimodal feature fusion classification model was proposed by using hip joint data (X-ray images, CT images, and clinical texts) in this study. Superior performance in hip disease classification was demonstrated by the model. It was demonstrated that the model not only surpassed single-modality approaches in accuracy, but also provided superior clinical net benefit and predictive reliability. The complementary strengths of different information sources were maximized by integrating clinical text with imaging features. Strong potential for early screening, precise classification, and treatment evaluation of hip diseases was shown by this approach. The clinical translation of artificial intelligence in orthopedic imaging diagnosis may be promoted as a result.

## Figures and Tables

**Table 1:** Demographic data of the internal dataset.

Demographic	N	Age (Years)
**Patients**	350	66.3 ± 12.3
**Gender**		
** -Females**	194	66.7 ± 13.2
** -Males**	156	65.2 ± 13.7
**Hips**	605	
** -Normal**	163	58.1 ± 19.0
** -Degenerative**	162	65.9 ± 13.1
** -Necrotic**	140	59.9 ± 14.6
** -FAI**	140	58.3 ± 14.3

**Table 2:** The baseline characteristics of the training set, validation set, and test set.

Characteristics	Training Set (N = 423)	Validation Set (N = 90)	Test Set (N = 92)	*p*
**Sex ratio (N): male/female**	195/228	35/55	45/47	0.458
**Age (years): mean ± SD**	59.7 ± 19.5	63.3 ± 13.8	60.5 ± 15.0	0.317

**Table 3:** Performance metrics for the classification model on the test set.

Classification	Precision	Recall	F1-Score	Sensitivity	Specificity	AUC
**Normal**	0.920	0.920	0.920	0.920	0.970	0.942
**Necrotic**	0.909	0.952	0.930	0.952	0.986	0.946
**Degenerative**	0.920	0.920	0.920	0.920	0.970	0.954
**FAI**	0.950	0.905	0.927	0.905	0.986	0.957

**Table 4:** Performance metrics of single-modality and multimodal fusion strategies on the internal test set.

Model	AUC	Sensitivity	Specificity	Precision	F1-Score
**CT**	0.867	0.855	0.885	0.860	0.857
**X**	0.843	0.830	0.860	0.835	0.832
**Clinical**	0.815	0.805	0.835	0.810	0.807
**X+CT**	0.916	0.900	0.930	0.905	0.902
**Clinical+CT**	0.886	0.870	0.900	0.875	0.872
**Clinical+X**	0.873	0.860	0.890	0.865	0.862
**Clinical+X+CT**	0.949	0.924	0.978	0.924	0.924

**Table 5:** Performance metrics for the classification model on the external validation set.

Classification	Precision (95% CI)	Recall (95% CI)	F1-Score (95% CI)	Sensitivity (95% CI)	Specificity (95% CI)	AUC (95% CI)
**Normal**	0.833 (0.550–0.960)	0.833 (0.550–0.960)	0.833 (0.600–0.950)	0.833 (0.550–0.960)	0.944 (0.780–0.990)	0.950 (0.820–1.000)
**Necrotic**	1.000 (0.650–1.000)	0.833 (0.550–0.960)	0.909 (0.700–0.990)	0.833 (0.550–0.960)	1.000 (0.850–1.000)	0.965 (0.830–1.000)
**Degenerative**	0.667 (0.600–0.900)	0.667 (0.600–0.900)	0.667 (0.550–0.880)	0.667 (0.600–0.900)	0.889 (0.700–0.980)	0.880 (0.862–0.980)
**FAI**	0.857 (0.550–0.980)	1.000 (0.700–1.000)	0.923 (0.750–0.990)	1.000 (0.700–1.000)	0.944 (0.780–0.990)	0.980 (0.780–1.000)

## Data Availability

The datasets analyzed during the current study are not publicly available due to patient privacy and ethical restrictions, but are available from the corresponding author on reasonable request.

## Abbreviations

The following abbreviations are used in this manuscript:

ONFH	Osteonecrosis of the femoral head
PACS	Picture Archiving and Communication System
DICOM	Digital Imaging and Communications in Medicine
Grad-CAM	Gradient-weighted Class Activation Mapping
DCA	Decision curve analysis
